# IL25 Enhanced Colitis-Associated Tumorigenesis in Mice by Upregulating Transcription Factor GLI1

**DOI:** 10.3389/fimmu.2022.837262

**Published:** 2022-03-14

**Authors:** Junxi Liu, Bingxiu Qian, Lin Zhou, Gang Shen, Yandan Tan, Siqi Liu, Zewei Zhao, Jianglin Shi, Weiwei Qi, Ti Zhou, Xia Yang, Guoquan Gao, Zhonghan Yang

**Affiliations:** ^1^Department of Biochemistry, Zhongshan School of Medicine, Sun Yat-Sen University, Guangzhou, China; ^2^Department of Biochemistry, Molecular Cancer Research Center, School of Medicine, Sun Yat-sen University, Shenzhen, China; ^3^Department of Laboratory Medicine, Third Affiliated Hospital of Sun Yat-sen University, Guangzhou, China

**Keywords:** IL25, colorectal cancer, AOM/DSS model, cancer stem cell, GLI1

## Abstract

Interleukin-25 (IL17E/IL25) plays a critical role in colitis and intestinal homeostasis. However, the expression and biological role of IL25 in colorectal cancer is not properly understood. In this study, we show that IL25 is mainly expressed by cancer stem cells in the colorectal cancer microenvironment. Genetic deletion of IL25 inhibited tumor formation and growth and prolonged survival in AOM/DSS-treated mice. IL25 stimulated cancer organoid and cancer cells sphere formation and prevented the tumor from chemotherapy-induced apoptosis. Mechanistically, IL25 upregulated stem cell genes LGR5, CD133, and ABC transporters *via* activating the Hedgehog signaling pathway. IL25 inhibited phosphorylation of AMPK and promoted GLI1 accumulation to maintain cancer stem cells. Moreover, IL25 expression was associated with poor survival in patients with metastatic colorectal cancer. Taken together, our work reveals an immune-associated mechanism that intrinsically confers cancer cell stemness properties. Our results first demonstrated that IL25, as a new potent endogenous Hedgehog pathway agonist, could be an important prognostic factor and therapeutic target for CRC.

## Introduction

Colorectal cancer (CRC) is the third most occurring malignancy and the third most common cause of cancer death worldwide (1). As the third-generation platinum drug, oxaliplatin is the first-line treatment for patients with metastatic colorectal cancer (mCRC) (2). Unfortunately, the 5-year survival rates for patients with metastasis are approximately 14% (3, 4). One of the major reasons for treatment failure and poor prognosis is drug resistance (5). Therefore, it is essential for us to clarify the mechanism of chemotherapy resistance and to develop approaches to prevent or reverse drug resistance for patients with mCRC.

Cancer stem cells (CSCs) play a major role in tumor growth, progression and can resist chemotherapeutic agents by increasing drug efflux ABC transporters and activating DNA repair machinery (6). It was noted that platinum drugs could be the substrates for selected ABC transporters ABCC2 and ABCC5 (7). Additionally, colorectal cancer cells could upregulate ABC transporters, such as ABCC2 to promote oxaliplatin resistance by activating the stem cell Hedgehog–GLI1 signal pathway (8). Furthermore, upregulation of CD44 and Lgr5 in CRC cells led to increased CSCs resistance to oxaliplatin and 5-FU (9). Besides, the decline of CSC markers, CD44, LGR5, and CD133 in the CRC organoids was more sensitive to oxaliplatin and 5-FU (10). Recently, it was suggested that differentiated cancer cells or progenitor cells can revert to CSCs through the cancer cell niche signals like WNT and EGF (11). However, it remains uncertain that the potent molecular lets the CRC cells differentiate into colorectal CSCs and gain the ability of chemotherapy resistance. Therefore, targeting some cancer niche secrete signals might be an effective strategy to eliminate colorectal CSCs and improve CRC patient prognosis.

Interleukin-25 (IL25), also known as IL-17E, is a member of IL-17 cytokine family, which includes IL-17A to IL-17F (12). It was found that IL25 was upregulated in DSS-induced colitis and played a significant role in intestinal parasitic infection and type 2 immunity (13–16). Furthermore, as the receptor of IL25, IL-17RB was found to be a marker of colorectal CSCs (17). In our previous study, we found that IL25 could promote liver cancer metastasis by inducing macrophages to secrete CXCL-10 (18). Besides, IL25 also promoted breast cancer liver metastasis by inducing macrophage M2 polarization (19). Conversely, Saori et al. reported that a high level of IL25 promoted IL-17RB^+^ breast cancer apoptosis (20). These studies imply that IL25 is closely related to cancer development, but the exact role of IL25 in colorectal cancer is unclear and controversial. Last but not least, outside of colitis, much less is known about the roles of IL25 in CRC.

Given that there is a strong correlation between colitis and CRC, we hypothesized that IL25 was continuously upregulated in CRC and promoted cancer development. In this study, we aimed to identify the effects of IL25 on decreasing colorectal cancer sensitivity to oxaliplatin by maintaining colorectal cancer stemness and the underlying mechanism.

## Materials and Methods

### Human Samples

A total of 49 cases of CRC tissue samples with survival information were collected from the Sun Yat-sen University Cancer Center. Informed consent of all patients has been obtained before surgery, and the use of medical records and histological sections has also been approved by the ethics committee in SYUCC. CRC tissue microarray (HColA150CS02, 74 cases) was purchased from the Shanghai Outdo Biotech (Shanghai, China). All procedures were performed under consensus agreements and following the Chinese Ethical Review Committee.

### Animals and Models for AOM-DSS-Induced CRC

Wild-type C57BL/6J mice were acquired from the center of laboratory animal of Sun Yat-sen University. The IL25 gene knockout (IL25KO) mice with C57BL/6J genetic background were acquired from the model animal research center of Nanjing University. All mice were maintained under 12 h light-dark cycles with a designed environmental temperature (21°C ± 1°C). All animal studies were conducted with the approval of the Institutional Animal Care and Use Committee (IACUC) of Sun Yat-sen University (approval number: SCXK2019-0209) meted with the China guideline of GB/T 35892-2018. This study was conducted following the ethical principles derived from the Declaration of Helsinki and the Belmont Report and was approved by the review board of Sun Yat-sen University (Guangzhou, China). Colorectal cancer (CRC) was induced by intraperitoneal injection of AOM (10 mg/kg; Sigma, A5486) combined with the Dextran sulfate sodium salt (DSS; MP, 160110) stimulus, resulting in tumor development restricted to the colon in mice as previously described (21). After injection of AOM on day 0, mice were given three rounds of a 2% DSS solution in their drinking water for 7 days starting on days 7, 28, and 49. Weight change during the experiment was calculated as the percent change in weight compared with the baseline measurement. The weight of the mice was monitored weekly. Mice were intraperitoneally injected with vehicle (5% glucose solution) or oxaliplatin (5 mg/kg once a week; Selleck, S1224) for two weeks.

### Cell Culture

The human CRC cell lines (SW48, CaCO2, LoVo, SW620, HT-29) and normal colonic epithelial cell lines CCD 841 were obtained from the American Type Culture Collection. Cell lines were authenticated by Cellcook Biotech. All cells were cultured and grown in DMEM supplemented with 10% FBS. After starvation for 6 h, CRC cells were treated with recombinant IL25 (R&D Systems; 8134-IL-025) in a dose-dependent manner. The GLI1 inhibitor, GANT-58 (MCE; HY-13282), the SMO inhibitor, Vismodegib (MCE; HY-10440), and the AMPK activator, Metformin (Sigma Aldrich, 1115-70-4) were added in serum-free DMEM medium for 24 h. For RNAi experiments, CRC cells were transfected with HiPerFect reagent (QIAGEN, #301705) using siRNA molecules (Generay, Shanghai, China).

### Cell Viability Assay

The viability of CRC cells was determined by Cell Counting Kit-8 (CCK-8) assay (Dojindo, CK04), following the instructions of the manufacturer. Briefly, 5,000–8,000 CRC cells per well were seeded in 96-well plates overnight. After starvation for 6 h, CRC cells were treated with or without IL25 for 36 h. Oxaliplatin (Selleck, S1224) was added in a dose-dependent manner for 48 h. Cell viability was measured by adding 10 μl of CCK-8 to each well. After 2 h of incubation at 37°C in a humidified incubator containing 5% CO_2_, the OD value was determined by absorbance at 450 nm using the Sunrise microplate reader (TECAN, Mäannedorf, Switzerland).

### Histology and Immunohistochemistry

Colorectal cancer tissue was fixed in 4% paraformaldehyde. Tissues were embedded in paraffin and sectioned by microtome. The slides were stained with hematoxylin and eosin (H&E) and immunohistochemical staining was conducted following standard protocol. Briefly, the sections were deparaffinized, rehydrated in a gradient of ethanol, and pretreated with 0.01 M citrate buffer (pH 6.0) through the high-pressure method. Then the sections were immersed in 3% H_2_O_2_ for 30 min to quench endogenous peroxidase. For IL25 immunohistochemistry, slides of various tissues were blocked with goat serum for 1 h. Subsequently, the slides were incubated with the following primary antibodies: IL25 (1:200; Novus Biologicals, NB100-56541) and LGR5 (1:100; Abcam, ab75732) antibody overnight at 4°C following incubation with HRP-conjugated secondary antibody for 1 h at room temperature and then stained with the DAB Horseradish Peroxidase Color Development Kit. Hematoxylin was used as counterstain. Sections were photographed through a slide scanner (Axio Scan. Z1, ZEISS). The degree of IL25 immunostaining was determined by the staining index (SI) which was reported elsewhere (22). The SI was calculated as the product of the grade of tumor cell proportions and the staining intensity score.

### Immunofluorescence

For immunofluorescence staining, slides were incubated with the following primary antibodies: GLI1 (1:100; Santa Cruz sc-515781), CD133(1:100; eBioscience 14-1331-82), LGR5 (1:100; Abcam, ab75732), DCAMKL1 (1:200; Abcam, ab31704), IL25 (1:200; Novus Biologicals, NB100-56541) overnight at 4°C, followed by staining with a mixture of secondary antibodies containing an Alex Flour 488-Donkey anti-rat IgG (H+L) (1:200; A21208) and an Alex Flour 594-Donkey anti-rabbit IgG (1:200; R37119) for 1 h at 37°C temperature. The cell nuclei were counterstained with 4’, 6-diamidino-2-phenylindole (DAPI) for 10 min at room temperature. The slides were observed with a confocal laser scanning microscope.

### Western Bolting

Tissues and cells were lysed in SDS buffer supplemented with 1 mM phenylmethanesulfonyl fluoride (Beyotime, ST506). The protein concentration was determined by the BCA protein assay kit (KeyGen, KGP902) and total cellular protein (30 ug) was subject to western blot analysis. The protein was transferred to 0.45 μm PVDF membrane (Millipore) and then the membranes were blocked with 7% of defatted milk in TBST (20 mM of Tris–HCl pH 7.4, 500 mM of NaCl, and 0.1% of Tween-20) for 1 h at room temperature. The membranes were incubated with the following primary antibodies: p-AMPKα (1:1,000; Thr172) (4188), AMPK (1:1,000; 2532), CD133 (1:1,000; 86781), BTRC (1:1,000; 4394) from Cell Signaling Technology; GLI1 (1:500; sc-515781), Smo (1:500; sc-166685), MRP2 (1:500; sc-59611), MRP5 (1:500; sc-376965), PTCH1 (1:500; sc-518102), IL-17E (1:500; sc-52933), SHH (1:500; sc-365112) CD133 (1:1,000; sc-365537) from SANTA CRUZ BIOTECHNOLOGY; LGR5 (1:1,000; ab75732), DCAMKL1 (1:1,000; ab31704) from Abcam; ALDH1A3 (1:1,000; Novus Biologicals, NBP2-15339), CD44 (1:1,000,15675-1-AP) and GAPDH (1:5,000,60004-1-Ig) from Proteintech Group. After incubation at 4°C overnight, membranes were probed with HRP-conjugated anti-rabbit IgG (Cell Signaling Tech, #7074) or anti-mouse IgG (Sigma-Aldrich, AP308P), then developed by ECL substrate (Merck Millipore) and visualized using the Bio-Rad ChemiDoc Touch Imaging System.

### Real-Time PCR

Total RNA from tissue or cells was extracted with TRIZOL reagent (Invitrogen, #15596026). RNA concentration was measured by the spectrometer. Approximately 1,000 ng total RNA was reverse transcribed into cDNA by PrimeScript reverse transcription reagent (TaKaRa, RR036A) following the instructions of the manufacturer. Real-time PCR analysis using SYBR Green PCR Mix (TakaRa, RR420A) was performed on the CFX96 PCR system (BioRad). ACTB was used as an internal normalization control. The normalized fold change of gene mRNA levels was calculated using the 2^−ΔΔCt^. The PCR primer sequences are listed in **Table S2**.

### Measurement of Free Fatty Acids and Cholesterol

Cholesterol (CHO) and free fatty acids (FFA) in plasma were measured by the TG assay kit (A111-1-1) and FFA assay kit (EFFA-100). All measurements were performed with standard manufacture protocol.

### Sphere Formation Assay

CRC cells were plated in 96-well ultralow attachment plates (Corning) in DMEM/F12 serum-free medium supplemented with 2% B27 (Thermo Scientific, 12587010), 20 ng/ml epidermal growth factor (EGF, Beyotime, P5552), 20 ng/ml basic fibroblast growth factor (bFGF, Beyotime, P6443) at a density of 1,000 viable cells/well. CRC cells were treated with recombinant IL25 (R&D Systems; 8134-IL-025). The GLI1 inhibitor, GANT-58 (MCE; HY-13282), the SMO inhibitor, Vismodegib (MCE; HY-10440), and the AMPK activator, Metformin (Sigma Aldrich, 1115-70-4) were added in sphere culture for 1 week. Tumor spheres (tight, spherical, nonadherent masses >50 µm in diameter) were counted, and their images were captured under an inverted microscope (Leica DMI4000B).

### Cancer Organoids Isolation

Intestinal fragments containing adenomas from WT or IL25KO AOM/DSS induced tumor or human colon tumor tissues were washed with PBS several times and incubated in Gentle cell dissociation reagent (STEMCELL, 07174) for 60 min on 37°C. intestinal adenomas were seeded in 24-well plates (500 crypts/fragments per 50 μl of Matrigel per well). The Matrigel was polymerized for 10 min at 37°C, and 500 μl/well basal culture medium (advanced Dulbecco’s modified Eagle medium/F12 supplemented with penicillin/streptomycin, 10 mmol/L HEPES, Glutamax, 1 × N2, 1 × B27 [all from Invitrogen], and 1 mmol/L N-acetylcysteine [Sigma]) (23).

### Ubiquitin Conjugated Assay

Colorectal cancer cells were treated with 10 μM MG132 (Merck, Germany) for the indicated treatment and times. The cells were washed with cold PBS and lysed in the radioimmunoprecipitation assay (RIPA) buffer (Beyotime, P0013D) with 1 mM PMSF (Beyotime, ST506), 1× phosphatase inhibitor (MCE, HY-K0021), and 1× cocktail (MCE, HY-K0010)) at 4°C overnight. After determining the total protein concentration, aliquots of equal amounts of protein were incubated with GLI1 antibody (1:100; sc-515781) overnight at 4°C. Next, Protein A/G PLUS-Agarose (SANTA CRUZ, sc-2003) were added and incubated for 4 h at 4°C. The beads were then centrifuged and washed with pre-cool basic RIPA buffer. After releasing with 2× SDS buffer, the precipitated proteins were subjected to western blot analysis with total cell lysates.

### Protein Half-Life Determination

CRC cells were pretreated with or without 50 ng/ml IL25 overnight and then incubated with cycloheximide (50 μg/ml; CHX, Sigma) for the indicated time and were analyzed by western blot analysis. The intensity of the bands was quantified using Image J software.

### Statistical Analysis

All data are presented as mean ± *SEMs*. The Student’s t-test was used to compare between two groups. One-way analysis of variance (ANOVA) or Two-way ANOVA was applied to compare more than two different groups. The relationships between IL25 expression and clinicopathological characteristics were determined using the chi-square test. Survival curves were plotted using the Kaplan–Meier method and compared using the log-rank test on GraphPad Prism 8.0 software. For each parameter of all data presented, NS (No Significance), *p  < 0.05, **p < 0.01, ***p < 0.001. p < 0.05 is considered significant.

## Results

### Elevated IL25 is Associated With Tumor Progression in CRC

**Figure 1:**
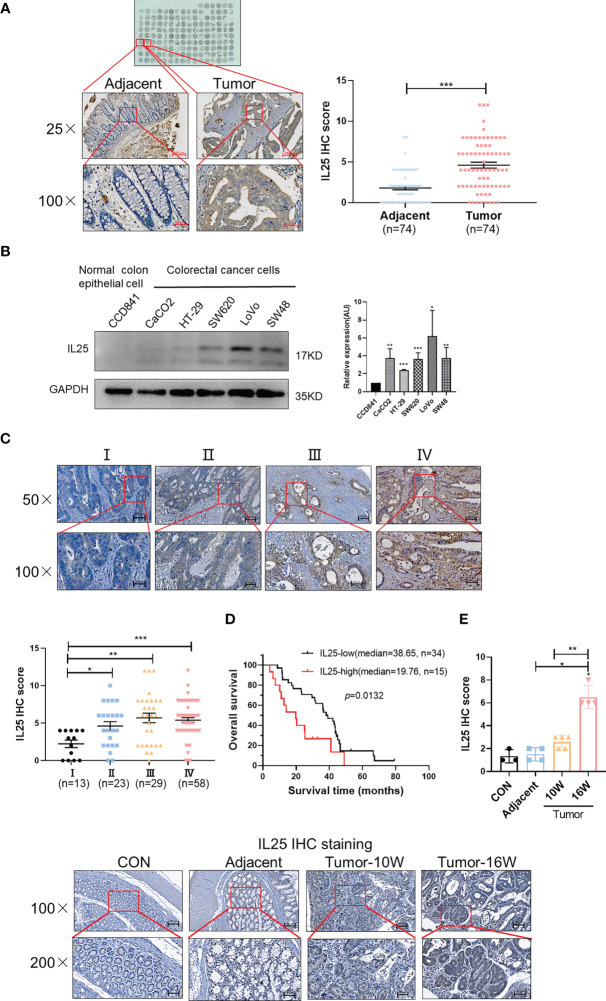
Overexpression of IL25 was found in CRC patients and predicts a poor prognosis. **(A)** Immunohistochemistry (IHC) staining of IL25 was performed in a tissue microarray consisting of 74 CRC tumor tissues and adjacent colon tissues (left). Statistical analysis of IL25 staining in adjacent specimens and CRC specimens (right). **(B)** Protein levels of IL25 were detected by Western blotting in normal intestinal cells (CCD841) and CRC cell lines (left). The right panel showed the quantitative analysis of the gray scan. The ImageJ software was used for gray scanning. **(C)** Representative images of IL25 IHC staining at different clinical stages (up). Correlation between IL25 expression and various clinical stages (down). **(D)** Overall survival curves of 49 CRC patients in correlation with intra-tumor IL25 IHC-scores. High IL25 expression was considered IHC-Score >6. The patients with CRC were divided into 2 groups according to the intra-tumor IL25 IHC-score: low group (n = 34), high group (n = 15). **(E)** Representative images of IL25 IHC staining from WT colon and AOM/DSS induced tumors on weeks 10 and 16 (down). Statistical analysis of IL25 staining in con colon, adjacent tissues, and AOM/DSS-induced CRC tissues (up). Data present as mean ± *SEM*; *p < 0.05, **p < 0.01, ***p < 0.001.

To explore the critical role of IL25 in CRC prognosis, we analyzed the GEO dataset GSE17258. Although their difference was not statistically significant (*p* = 0.1174), it was shown that the OS of the IL25-high expression group was even 29.1 months shorter than that of the low expression group in Stage III & IV patients (**Figures S1A, B**). To further verify the crucial role of IL25 in the progression of CRC, we analyzed the protein level of IL25 by CRC Tissue Microarray. Impressively, compared with adjacent specimens (IHC-Score = 1.810), the expression of IL25 was remarkably elevated in CRC specimens (IHC-Score = 4.608; **Figure 1A**). Similar to the results in CRC tissues, IL25 expression was augmented in CRC cells compared with normal colon epithelial cell lines (**Figure 1B**). Meanwhile, we retrospectively studied the medical records of 123 CRC patients and identified that IL25 expression increased along with the progression of CRC clinical stages (**Figure 1C**). The 5-year OS rate of the IL25-high group (n = 15; median = 19.76) was significantly lower than that of the IL25-low group in Stage IV patients (n = 34; median = 38.65; *p* = 0.0133) (**Figure 1D**). Taken together, the upregulation of IL25 was closely relevant with progression and poor prognosis of CRC. To model colitis-associated colon cancer, we established wild-type female C57BL/6J (WT) AOM/DSS-induced mouse CRC models. Impressively, compared with control and adjacent colon specimens (IHC-Score = 1.33 and 1.50), the expression of IL25 had a rising trend in WT 10-week tumor (IHC-Score = 2.6) and was remarkably elevated in WT 16-week tumor (IHC-Score = 6.5) (**Figure 1E**).

### IL25 Promoted the Progression of Colitis-Associated Cancer (CAC) *In Vivo*

**Figure 2:**
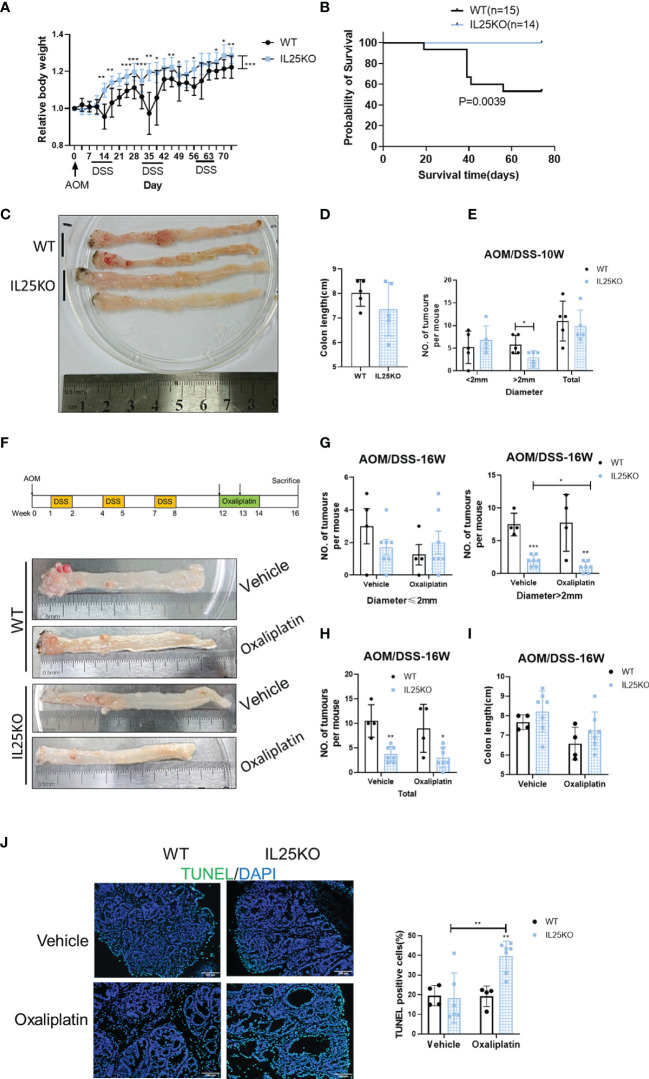
Genetic deletion of IL25 inhibited the progression of the Colitis-Associated Cancer (CAC) Model. IL25KO or WT control mice were given an intraperitoneal injection of AOM on day 1, 2.5% DSS in drinking water for 7 days starting on days 7, 28, and 42, and euthanized on days 70 and 112. **(A)** Bodyweight change during colitis-associated colorectal cancer with AOM/DSS as a percentage of initial weight. **(B)** Overall survival curves of WT and IL25KO mice. **(C)** Representative images of colonic tumors from WT and IL25KO mice in 10 weeks. **(D)** Total number and size of tumors along the colon in WT (n = 5) and IL25KO (n = 5). **(E)** Colon length in mice treated with the indicated treatment in 10 weeks. **(F)** Effect of oxaliplatin on WT and IL25KO AOM-DSS-induced CRC mouse models. The colon was removed, cut lengthwise, washed with PBS, and digitally photographed. **(G)** Size of individual tumors along the colon in WT treated with vehicle (n = 4) or oxaliplatin (n = 4) and IL25KO treated with vehicle (n = 7) or oxaliplatin (n = 7). **(H)** Total number and size of tumors. **(I)** Colon length in mice treated with the indicated treatment on 16 weeks. **(J)** Representative immunofluorescent stains for TUNEL in colonic sections from WT and IL25KO treated with vehicle or oxaliplatin (left). Statistical analysis of Tunel staining in WT and IL25KO tumors (right). Data present as mean ± *SEM*; *p < 0.05, **p < 0.01, ***p < 0.001.

To further verify the decisive role of IL25 in the progression of CRC, we also treated IL25KO mice with AOM/DSS to induce CRC. During the challenge, female IL25KO mice exhibited less weight loss and a higher survival rate than WT controls (**Figures 2A, B**). During AOM and DSS challenge, genetic deletion of IL25 had retarded the development of colitis-associated cancer (**Figures 2C–E**). While total tumor numbers were no obvious change, the tumor size of IL25KO mice was smaller than WT in 10-week (**Figure 2D**). We further examined the efficacy of oxaliplatin in WT and IL25KO AOM/DSS-induced CRC models (**Figure 2F**). Surprisingly, IL25 deletion significantly decreased tumor numbers and size in 16-week and the tumors of IL25KO mice were further attenuated by oxaliplatin, while there was only a decreased trend in tumors of WT mice whose diameters were smaller than 2 mm (**Figures 2F–H**). Furthermore, enhanced apoptosis showed by Tunel staining was observed in tumors of IL25KO mice (**Figure 2J**). Through GSEA gene enrichment analysis, we found that ABCC2 and ABCC5 were significantly increased in IL25-high CRC and had a positive correlation with IL25 (**Figures S1C, D**). By western blotting, we found that IL25 associated with ABCC2 and ABCC5 were increased after oxaliplatin treatment (**Figure S2C**). *In vitro*, HT-29 cells treated with IL25 were less sensitive to the oxaliplatin treatment (**Figure S2D**). To further validate whether IL25 regulates ABC transporters, real-time PCR was utilized to detect the change of ABC transporters which play essential roles in drug resistance. Impressively, we found that ABCC2 and ABCC5 mRNA and protein levels were significantly upregulated by IL25 (**Figures S2E, F**). Whereas, ABCC2 and ABCC5 were downregulated in IL25 silenced LoVo cells (**Figures S2G, H**). Moreover, IL25 silenced LoVo cells were more sensitive to the oxaliplatin treatment than the cells transfected with NC control siRNA (**Figure S2I**). Together, these data suggested that IL25 decreased the sensitivity of oxaliplatin in CRC by upregulating ABC transporters.

### IL25 Maintained Colorectal Cancer Stemness

**Figure 3:**
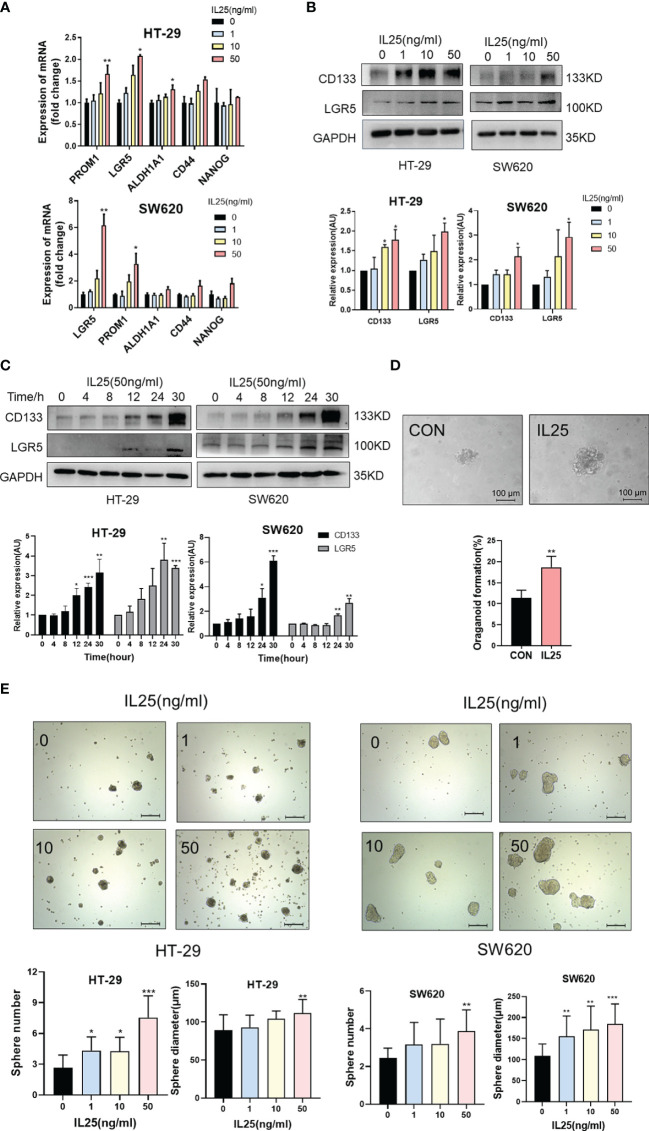
IL25 promoted the stemness of CRC cells. **(A)** The expression levels of CSC markers, namely, CD133 and LGR5, were examined in HT-29 and SW620 cells treated with recombinant IL25 in a concentration-dependent manner by RT-PCR. **(B, C)** The expression levels of CD133 and LGR5 were examined in HT-29 and SW620 cells treated with recombinant IL25 in a concentration and time-dependent manner by Western blotting. **(D)** Frequency (down) and representative day-7 images (up) of human colon adenomatous organoids treated with/without IL25 (50 ng/ml). **(E)** Sphere formation analysis of HT-29 and SW620 cells treated with recombinant IL25 in a concentration-dependent manner. Representative images (up) and the mean numbers and sphere size (down) of spheres are shown. Scale bar, 200 μm. Data present as mean ± *SEM*; *p < 0.05, **p < 0.01, ***p < 0.001.

To further clarify the role of IL25 in CRC development, we performed Ki67 immunohistochemical staining in WT and IL25KO tumors slices. Since the proliferation, apoptosis, and colon length had no difference between WT and IL25KO (**Figures 2D, I, J** and **Figure S2A**), we found IL25 and DCLK1 were upregulated in oxaliplatin-treated tumors (**Figure S2C**). We also found DCLK1^+^ or CD133^+^ cells could secrete IL25 (**Figures S3E, F**). This finding indicated that IL25 may be involved in CSCs maintaining. Therefore, we analyzed the RNA sequence in colorectal cancer in the GEO dataset GSE17538. The gene signatures of cancer stem cells were positively correlated with IL25 expression (**Figure S1D**). In addition, LoVo spheres sorted by sphere formation also had higher IL25 expression than control LoVo cells (**Figure S3D**). Then we analyzed the mRNA levels of stemness-related markers in IL25 treated HT-29 and SW620 cells, which showed that expression of stemness-related markers, especially, CD133, LGR5, and OCT-4 were elevated in IL25 treated CRC cells (**Figure 3A** and **Figure S3G**). Meanwhile, western blotting results revealed that CD133 and LGR5 were upregulated in IL25 treated CRC cells in a dose and time-dependent manner (**Figures 3B, C**). To determine whether IL25 affects the frequency of colorectal CSCs, a cancer organoid model was established from freshly isolated primary tumors from colon cancer patients, which showed that IL25 increased the frequency of cancer organoid formation (**Figure 3D**). Then, the sphere formation assays were carried out to inspect the influence of IL25 on the self-renewal capability of CRC cells. After a 7-day culture, the numbers and sizes of spheres in the IL25 treated CRC cells were more remarkable than that of the control group (**Figure 3E**).

**Figure 4:**
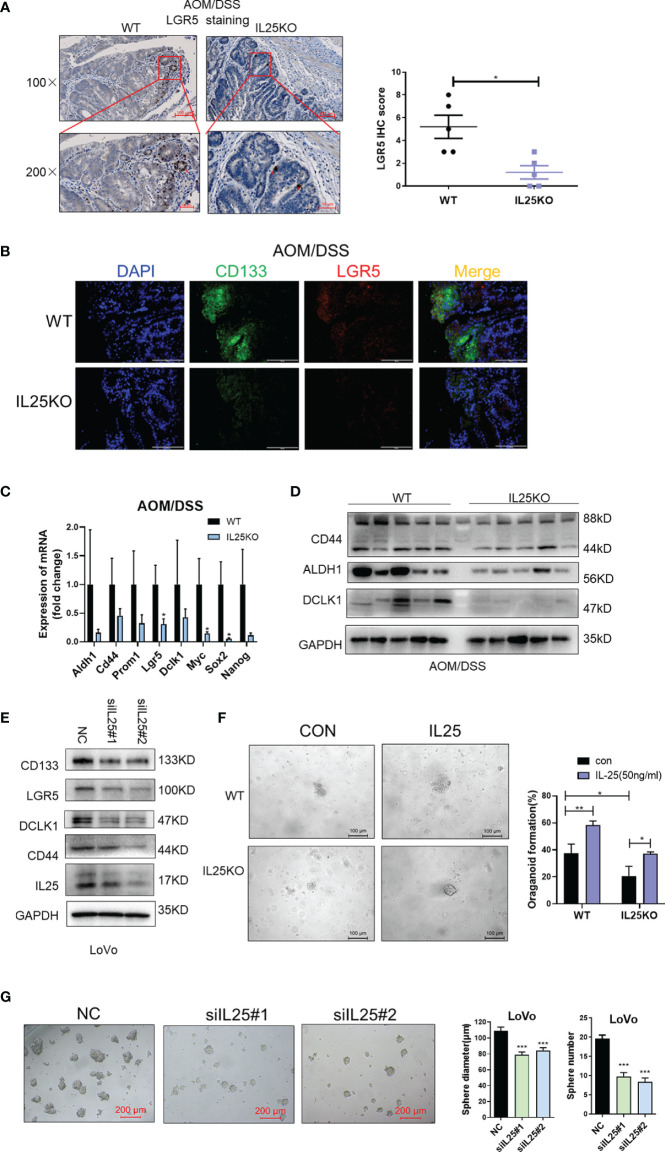
IL25 Deficiency Induced loss of CRC stemness. **(A)** Representative images of LGR5 IHC staining (left). Statistical analysis of LGR5 staining in WT and IL25KO tumors (right). **(B)** Stem cell markers CD133 and LGR5 expression were detected by multiplexed fluorescence staining in the WT and IL25KO AOM-DSS-induced CRC mouse models. The representative images show the expression of LGR5 (red), DAPI (blue), CD133 (green). **(C)** The expression levels of stem cells markers were examined in WT and IL25KO tumors by RT-PCR. **(D)** The expression levels of CSC markers, namely, DCLK1, ALDH1, and CD44, were examined in tumor tissues by Western blotting. **(E)** Western blotting of Stem cell markers levels in LoVo cells following IL25 silencing. **(F)** Frequency (right) and representative day-7 images (left) of adenomatous organoids from WT and IL25KO mice treated with/without IL25 (50 ng/ml). **(G)** Sphere formation analysis of LoVo cells following IL25 silencing. Representative images (left) and the mean numbers and sphere size (right) of spheres are shown. Data present as mean ± *SEM*; *p < 0.05, **p < 0.01, ***p < 0.001.

On the contrary, LGR5 and CD133 positive cells were decreased in IL25KO tumors (**Figures 4A, B**) and the expression of stemness-related markers, especially, LGR5, Myc, and Sox2 were downregulated in IL25KO tumors (**Figure 4C**). Meanwhile, we observed the stemness-related markers, CD44, ALDH1, DCLK1 were downregulated in IL25KO AOM/DSS induced tumors (**Figure 4D** and **Figure S3H**). *In vitro*, silencing IL25 reduced the stemness-related markers, CD133, LGR5, CD44, ALDH1, and DCLK1 (**Figure 4E** and **Figure S3I**). Moreover, deletion of IL25 decreased the frequency of cancer organoid formation, which was reversed by IL25 (**Figure 4F**). Notably, knockdown of IL25 in LoVo spheres substantially reduced the numbers and sizes of the formed spheres (**Figure 4G**). Collectively, these results indicated that IL25 maintained the stemness of CRC cells.

### IL25 Mediates Stemness Through the Activation of the Hedgehog Signaling

**Figure 5:**
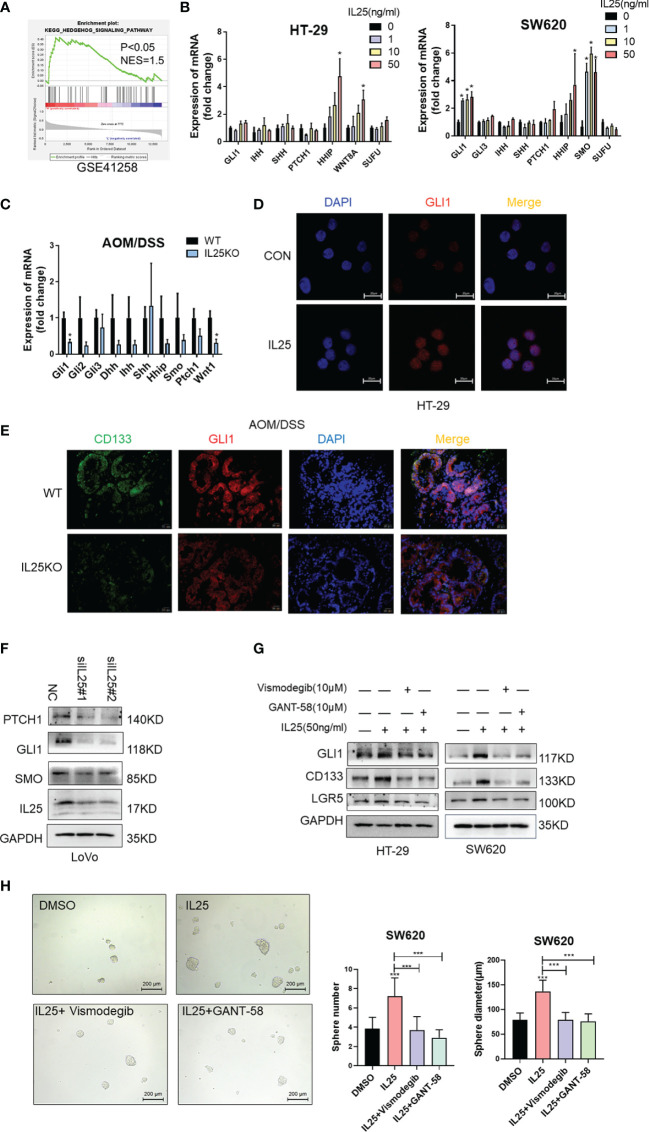
IL25 promoted stemness *via* the Hedgehog signaling pathway in CRC cancer. **(A)** GSEA for Hedgehog signaling pathway (nominal *p < *0.05) in IL25 high expression colorectal cancer compared with IL25 low expression colorectal cancer from GSE41258. **(B)** The expression levels of Hedgehog signaling genes were examined in HT-29 and SW620 cells treated with recombinant IL25 in a concentration-dependent manner by RT-PCR. **(C)** The expression levels of Hedgehog signaling genes were examined in WT and IL25KO AOM/DSS-induced CRC cancer tissues by RT-PCR. **(D)** GLI1 expression was detected by immunofluorescence staining in HT-29 cells treated with or without 50 ng/ml IL25. **(E)** GLI1 and CD133 expression were detected by multiplexed fluorescence staining in the WT and IL25KO AOM/DSS-induced tumor tissues. Representative images show the expression of GLI1 (red), DAPI (blue), and CD133 (green). **(F)** Western blotting of Hedgehog signaling genes in LoVo cells following IL25 silencing. **(G)** Western blotting of GLI1 and CD133 in HT-29 and SW620 cells treated with SMO inhibitor Vismodegib and GLI1 inhibitor GANT-58 following IL25 treatment. **(H)** Sphere formation analysis of SW620 cells treated with SMO inhibitor Vismodegib and GLI1 inhibitor GANT-58 following IL25 treatment. Representative images (left) and the mean numbers and sphere size (right) of spheres are shown. Data present as mean ± *SEM*; *p < 0.05, ***p < 0.001.

To identify pathways that may regulate CRC stemness, gene set enrichment analysis (GSEA) was carried out, comparing the IL25-high group to IL25-low group from GEO dataset GSE17538 and GSE41258. Our analysis demonstrated the transcriptome of the IL25-high group to be enriched in gene sets associated with the Hedgehog signaling pathway (**Figure 5A** and **Figures S1E, F**). Meanwhile, mRNA transcription of the downstream targets of Hedgehog signaling, namely, GLI1, SMO, HHIP, WNT8A, were upregulated in IL25 treated CRC cells (**Figure 5B**). Meanwhile, GLI1 and WNT1 were downregulated in IL25KO tumors (**Figure 5C**). Next, IL25 dramatically increased the GLI1 nuclear signals showed by immunofluorescence assays whereas IL25 deletion reduced GLI1 nuclear translocation (**Figures 5D, E**). By western blotting, it was shown that GLI1, PTCH1, and SMO declined in IL25 knockdown LoVo cells (**Figure 5F** and **Figure S4A**). To further delineate the role of GLI1-dependent Hedgehog signaling in IL25 mediated stemness, CRC cells were treated with small molecule inhibitors of Hedgehog (the SMO inhibitor vismodegib and GLI1 inhibitor GANT-58). Inhibition of SMO and GLI1 blocked the increase of CD133 and LGR5 and spheres formation mediated by IL25 (**Figures 5G, H** and **Figure S4B**). These results indicated that IL25 enhanced the Hedgehog signaling by regulating GLI1.

### IL25 Upregulates GLI1 Through Inhibiting p-AMPK

**Figure 6:**
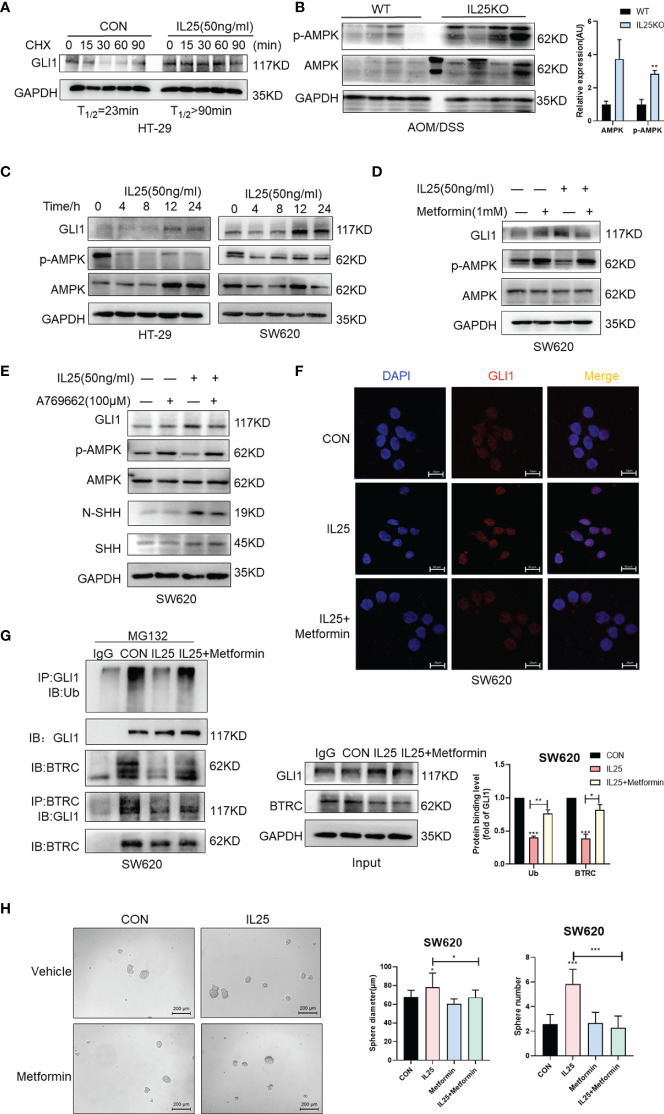
IL25 upregulated GLI1 by inhibiting p-AMPK. **(A)** HT-29 cells were treated with cycloheximide (CHX, 50 μg/ml) for the indicated time, and cell lysates were analyzed by Western blotting with the indicated antibodies. **(B)** Western blotting of p-AMPK and AMPK in the WT and IL25KO AOM/DSS-induced tumor tissue. **(C)** The expression levels of GLI1, p-AMPK, and AMPK were examined in HT-29 and SW620 cells treated with recombinant IL25 in a time-dependent manner by Western blotting. **(D, E)** Western blotting of GLI1, p-AMPK, and AMPK in SW620 cells treated with AMPK activator A769662 and Metformin following IL25 treatment. **(F)** GLI1 expression was detected by immunofluorescence staining in SW620 cells treated with AMPK activator Metformin following IL25 treatment. **(G)** SW620 cells were treated with 10 μM MG132 and then incubated with or without 50 ng/ml recombinant IL25 and 1 mM Metformin, then immunoprecipitated with GLI1 antibody. GLI1 ubiquitination was determined using an anti-ubiquitin antibody. IP, immunoprecipitation. **(H)** Sphere formation analysis of SW620 cells treated with AMPK activator Metformin following IL25 treatment. Representative images (left) and the mean numbers and sphere size (right) of spheres are shown. Data present as mean ± *SEM;* *p <0.05, **p <0.01, ***p <0.001.

To investigate whether IL25 affects the protein stability of GLI1, we measured the half-life of GLI1 by using cycloheximide (CHX), which blocks protein synthesis *in vitro*. The GLI1 degradation was decelerated in the presence of IL25 with CHX, while GLI1 had a half-life of approximately 23 min (**Figure 6A**). To clarify whether IL25 increases GLI1 by inhibiting p-AMPK, we detected p-AMPK by Western blotting. IL25KO tumors showed higher p-AMPK levels than wild-type mice (**Figure 6B**). Moreover, it was shown that IL25 inhibited p-AMPK before GLI1 accumulation (**Figure 6C** and **Figure S4C**), which was reversed by adding AMPK activator A769662 and metformin (**Figures 6D, E** and **Figures S4D**, **S5E**). Besides, GLI1 nuclear signals increased by IL25 were also blocked by metformin (**Figure 6F**). Furthermore, MG132 treatment with IL25 significantly decreased GLI1 polyubiquitination levels which were reversed by metformin (**Figure 6G**). Inhibition of p-AMPK blocked increased spheres and organoid formation mediated by IL25 (**Figure 6H** and **Figure S4E**).

**Figure 7:**
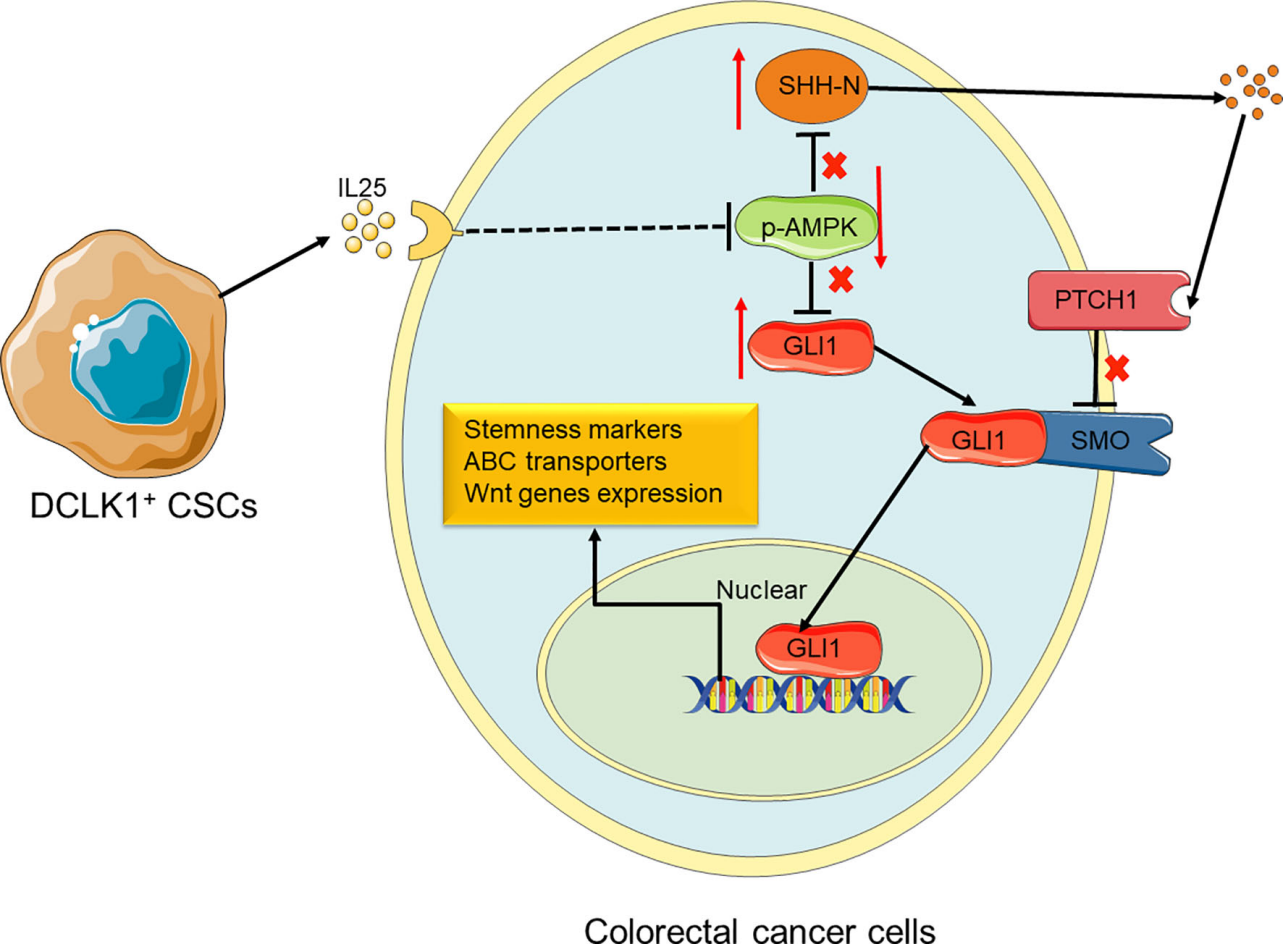
Proposed model for the roles and functions of IL25 in promoting CRC stemness. IL25 can inhibit p-AMPK and lead to GLI1 accumulation. It also stimulates SHH secretion by the means that minus times minus equals plus, thereby GLI1 binding with SMO. In nuclear, GLI1 can promote stem cell markers and ABC transporters expression. Therefore, CRC cells gain the ability of stemness and oxaliplatin resistance.

Additionally, we also found that IL25 increased N-SHH in a time-dependent manner (**Figure S5A**). Furthermore, IL25 upregulated HMGCR expression and cholesterol (**Figures S5B–D**). Similarly, IL25 promoted SHH-N through p-AMPK inhibition, which was reversed by activating p-AMPK (**Figure 6E**). IL25 increased SMO, GLI1, CD133, and SHH-N expression, which was reversed by silencing SHH receptor PTCH1 (**Figures S5E, F**). These results demonstrated that IL25 could activate the Hedgehog signaling pathway by inhibiting p-AMPK.

## Discussion

In this study, we identified that IL25 was strikingly elevated in the tissue of CRC patients and AOM/DSS-induced tumors, and high IL25 expression in CRC tissue was negatively correlated with survival rate. IL25 treated CRC cells substantially enhanced the expression levels of CD133 and LGR5, the formation of tumor organoid and sphere, thus decreasing the sensitivity to oxaliplatin of CRC cells. Consistently, silencing or deletion of IL25 *in vitro* and *in vivo*, decreased the formation of tumor organoid and sphere formation, thus enhancing the sensitivity to oxaliplatin of tumor. We first demonstrated that IL25 maintained CRC stemness through inhibiting p-AMPK and increased GLI1. This study provides evidence of a novel treatment strategy for CRC stemness by inhibition of IL25 centered pathway in CRC patients (**Figure 7**).

In the past, IL25 was considered to induce strong type 2 immunity in the gastroenterological tract characterized by increased expression of IL-5, IL-13, etc. (24). However, our previous research showed that deletion of IL25 in C57BL/6J protected from DSS-induced colitis. Endogenous IL25 acts as a pro-inflammatory factor in DSS-induced colitis by upregulated IL33 but not IL13 (25). AOM/DSS-induced CRC models resemble many aspects of the pathogenic process of human ulcerative colitis and CAC (26–28). A previous study showed that genetic deletion of IL25 did not affect tumor burden caused by AOM and 2 cycles DSS treatment in BALB/c mice (29). However, in our study, with the progression of CRC by AOM and 3 cycles DSS treatment, the numbers and volumes of tumors were significantly decreased in 16 weeks IL25KO C57BL/6J mice. It is known that mice in C57BL/6J background are prone to Th1 immunity, whereas mice in BALB/c background are biased toward type 2 immunity. Genetically deficient of IL13 in C57BL/6J mice are more susceptible to acute DSS-induced colitis. On the contrary, IL13KO in a BALB/c background showed reduced severity of DSS-induced colitis (25). It is likely that IL25 promotes colitis in an IL33 dependent manner on C57BL/6J, but promotes IL13 in BALB/c, which leads to the differences between the disparate strains. Our previous study showed that IL25 was not directly affecting the growth, apoptosis, or migration in HCC, but promoted macrophages secret CXCL10 and led to cancer metastasis (18). Similarly, we also discovered that the proliferation and apoptosis were not different between WT and IL25KO tumors. Cancer stem cells play an important role in tumor initiation, propagation, and therapy resistance, which are thought to be quiescent and more resistant to chemotherapy (6). In chronic myeloid leukemia (CML), CSCs were quiescent and more resistant to chemotherapy (30). Moreover, the undifferentiated tumor with stem cell signaling overexpression is associated with lower immune infiltration and downregulated programmed cell death 1 ligand 1 (PD- L1) signaling predicted a poor response to immunotherapy (31–33). Continuing studies elucidate that CSCs recruit immune cells to modulate a favorable microenvironment through secreting chemokines, cytokines, and inflammatory factors. At the same time, the topology and dynamic behavior of CSCs are sculpted by chemokines, cytokines, and inflammatory factors such as IL-6, IL-8, IL-22, and IL-33 (11, 34, 35). In addition, a previous study found that IL25 expression in breast cancer was a positive correlation with infiltrating CD4^+^T cells and macrophages, whereas IL25 blockade decreased type 2 T cells and macrophages in the primary tumor microenvironments and inhibited lung metastasis (19). Moreover, IL25 could promote proliferation and sustain self-renewal of NANOG positive hepatocellular carcinoma by activating NF-κB and JAK/Stat3 pathways (36). These data suggested that IL25 may be a key linkage between CSCs and the tumor microenvironment thus promoting CRC development.

DCLK1^+^ tuft cells which are the main producers of IL25 in the intestine had stem cell properties and played an important role in colitis and CRC initiation (37). In the intestine, tuft cells are the main producers of IL25 in the steady state. Besides, in mice DSS-induced APC lacking colonic adenocarcinoma, long-lived intestinal tuft cells serve as colon cancer-initiating cells (37). Moreover, tuft cells marker DCLK1 was especially expressed in intestinal tumor stem cells, whereas, it was hardly expressed in the colon steady stage (38, 39). However, how tuft cells drive tumor initiation and development remains unknown. Our data showed that IL25 was secreted by CD133 or DCLK1 positive cells (**Figures S3E, F**). At the same time, we found DCLK1 and IL25 were upregulated in tumors after oxaliplatin injection (**Figure S2C**). A previous study showed that high-fat diet increased intestinal stem cells and progenitor cells (40). To our surprise, free fatty acid and cholesterol were increased in AOM/DSS-induced mice serum which was injected with oxaliplatin (**Figures S3A, B**). Moreover, oleic acid could upregulate DCLK1 and IL25 expression *in vitro* (**Figure S3C**). These data suggested that oxaliplatin increased DCLK1^+^ CSCs through lipid. Read in conjunction, IL25 may play a curial role in chemotherapy resistance of CRC derived by tuft or cancer stem cells. On the other hand, CSCs markers are commonly shared by normal stem cells. Thus, therapies that target these markers may cause severe injury to normal colonic tissues. Our data demonstrated that IL25 was especially expressed in colorectal CSCs and is essential for maintaining cancer stemness. Targeting the IL25 signaling pathway may offer the potential to upgrade chemotherapy efficiency for colorectal cancer without damaging normal stem cells.

Our study comprehensively explored the mechanism involved in the process of IL25 enhanced CRC stemness. As GSEA analysis, we found IL25 could activate the Hedgehog signaling pathway. The Hedgehog signaling pathway plays a critical role in tissue-patterning during embryonic development and the repair of normal tissues, and cancer development (41). GLI1, GLI2, and GLI3 are the key transcription factors of the Hedgehog signaling pathway (42). However, we found GLI2 and GLI3 were barely detectable in colorectal cancer cells. A previous study showed that GLI1 was mainly phosphorylated by PKA, GSK3β, CK and degraded mediated by Ub (43). In the presence of SHH ligand, SMO was associated with β-arrestin and the microtubule motor KIF3A and prevented GLI1 from degradation (44). However, a recent study showed that p-AMPK could phosphorylate GLI1, thereby promoting GLI1 degradation, even in the presence of SHH ligand (45). Meanwhile, GLI1 could promote chemoresistance by upregulating ABC transporters in CRC cells (8). However, the relationship between IL25 and GLI1 had never been reported. It was also found that inhibition of p-AMPK could upregulate the key enzyme of cholesterol synthesis HMGCR and promote SHH CHLation, then increased N-SHH activated GLI1 binding target genes (46). Our research first identified that IL25 could activate the Hedgehog signaling pathway by inhibiting p-AMPK, and the induction of CRC stemness mainly depends on increasing SHH and nuclear GLI1. The most clinical inhibitor targeting the Hedgehog pathway is vismodegib, which was approved by the US FDA in 2012 and the European Medicines Agency (EMA) in 2013 for the treatment of metastatic basal cell carcinoma (BCC) or locally advanced BCC in patients who are not candidates for surgery or radiotherapy (47). However, the addition of vismodegib to combination treatment with FOLFOX (5-fluorouracil [5-FU], folinic acid and oxaliplatin) or FOLFIRI (5-FU, folinic acid, and irinotecan) chemotherapy plus bevacizumab did not increase PFS or the overall response rate (ORR) in metastatic colorectal cancer (48). Our research provides a novel strategy for the treatment of augmented levels of IL25 metastatic CRC by targeting the Hedgehog pathway by vismodegib.

Upregulation of IL25 may lead to inflammatory disorders such as atopic dermatitis, psoriasis, or asthma (49). Encouragingly, IL25 monoclonal antibody XKH001 was the first IL25 inhibitor which was approved by the FDA in 2021 for the clinical trial of inflammatory associated diseases, and IL25 blockade inhibited lung metastasis in breast cancer (19). Based on our findings, injection of IL25 neutralizing antibody would reduce the tumor in AOM/DSS treated mice. In the future, IL25 inhibition will be a new clinical strategy for colitis-associated cancer. In conclusion, our research first demonstrated that IL25 was elevated in CRC and inhibited p-AMPK, and upregulated GLI1, thereby maintaining stemness. This study indicated that IL25 would be a significant prognostic factor. Targeting IL25 by neutralizing antibodies and Hedgehog signaling pathway may improve chemotherapy efficacy and serve as a potential treatment for CRC patients.

## Data Availability Statement

The original contributions presented in the study are included in the article/**Supplementary Material**. Further inquiries can be directed to the corresponding authors.

## Ethics Statement

The studies involving human participants were reviewed and approved by the Sun Yat-sen University Cancer Center. The patients/participants provided their written informed consent to participate in this study. The animal study was reviewed and approved by the Institutional Animal Care and Use Committee (IACUC) of Sun Yat-sen University (approval number: SCXK2019-0209).

## Author Contributions

JL, BQ performed the experiments and wrote the first draft of the manuscript. LZ and GS revised the manuscript and performed the animal breeding and identification experiments. YT, SL, ZZ, JS: worked together on the collection of associated data and their interpretation and collaborated with all other authors. WQ and TZ discussed the data. ZY, XY and GG: conceptualization, supervision. All authors read and approved the final manuscript.

## Funding

This research was funded by the National Nature Science Foundation of China (Grant numbers: 81570764, 81872165, and 82070888); the National Key R&D Program of China (Grant number: 2018YFA0800403); the Guangdong Provincial Key R&D Program (Grant number: 2018B030337001); the Key Project of Nature Science Foundation of Guangdong Province, China (Grant number: 2019B1515120077); the Guangzhou Science and Technology Project (Grant number No: 201807010069), the Shenzhen Science and Technology Project (Grant number: JCYJ20190807154205627), the Guangdong Natural Science Fund (Grant numbers: 2020A1515010365, 2021A1515010434), the Guangdong Provincial Key Laboratory of Precision Medicine and Clinical Translation Research of Hakka Population (Grant number No: 2018B030322003KF01), the Key Sci-Tech of Hakka Population (Grant number No: 2018B030322003KF01), Key Sci-Tech. The funders had no role in study design, data collection, analysis, decision to publish, or preparation of the manuscript.

## Conflict of Interest

The authors declare that the research was conducted in the absence of any commercial or financial relationships that could be construed as a potential conflict of interest.

## Publisher’s Note

All claims expressed in this article are solely those of the authors and do not necessarily represent those of their affiliated organizations, or those of the publisher, the editors and the reviewers. Any product that may be evaluated in this article, or claim that may be made by its manufacturer, is not guaranteed or endorsed by the publisher.

## Supplementary Material

Supplementary Figure 1 GEO dataset analysis. **(A, B)** Overall survival analysis of CRC patients from GEO dataset GSE17538 in correlation with tumor IL25 expression. **(C)** Heat map showing core enrichment hallmark ABC transporters signaling genes in IL25 high expression colorectal cancer compared with IL25 low expression colorectal cancer from GSE17538. **(D)** Correlation analysis between IL25 and ABC transporters or stem cell markers from GSE17538. **(E)** Heat map showing core enrichment hallmark Hedgehog signaling genes in IL25 high expression colorectal cancer compared with IL25 low expression colorectal cancer from GSE41258. **(F)** GSEA for Hedgehog signaling pathway in IL25 high expression colorectal cancer compared with IL25 low expression colorectal cancer from GSE17538.

Supplementary Figure 2 IL25 expression was positively associated with chemotherapy drug transporters expression. **(A)** Representative images of Ki67 IHC staining. **(B)** Representative images of hematoxylin and eosin (H&E) from tumors in WT and IL25KO mice. **(C)** The expression levels of ABCC2, ABCC5, and IL25 were examined in tumor tissues by Western blotting. **(D)** HT-29 cells cultured with or without IL25 (50 ng/mL) for 24 hours and were subsequently exposed to oxaliplatin for 48 hours in a concentration-dependent. The cell viability was determined by the CCK-8 assay. **(E, F)** The expression levels of ABC transporters in HT-29 and SW620 cells treated with recombinant IL25 in a concentration-dependent manner by RT-PCR and Western blotting. **(G)** Silencing effect of various siRNA of IL25 in LoVo cells. **(H)** Western blotting of ABCC2 and ABCC5 in LoVo cells following IL25 silencing. **(I)** LoVo cells treated with oxaliplatin for 48 hours in a concentration-dependent following IL25 silencing. Data present as mean ± *SEM*; **p* < 0.05, ***p* < 0.01, ****p* < 0.001.

Supplementary Figure 3 IL25 was positively associated with stem cell genes expression. **(A, B)** Serum cholesterol and free fatty acid content in mice treated with the indicated treatment. **(C)** CRC cells were treated with 100 and 200μM oleic acid for 24h and cell lysates were analyzed by Western blotting with the indicated antibodies. **(D)** LoVo spheres were sorted from sphere formation assays by passaging spheres three times. LoVo cells and LoVo spheres lysates were analyzed by Western blotting. **(E)** IL25 and DCLK1 expression were detected by multiplexed fluorescence staining in the CRC patients and WT AOM/DSS-induced tumor tissues. The representative images show the expression of DCLK1 (red), DAPI (blue), IL25 (green). **(F)** IL25 and CD133 expression was detected by multiplexed fluorescence staining in the WT colon and WT AOM/DSS-induced tumor tissues. The representative images show the expression of IL25 (red), DAPI (blue), CD133 (green). **(G)** The expression levels of pluripotent stem cells markers, including NANOG, SOX2, MYC, POU5F1 were examined in HT-29 cells treated with recombinant IL25 in a concentration-dependent manner by RT-PCR. **(H)** Quantitative analysis of the western blotting of stem cells markers was examined in WT and IL25KO tumors. **(I)** Quantitative analysis of the western blotting of stem cells markers was examined in LoVo cells following IL25 silencing. Data present as mean ± *SEM*; **p* < 0.05, ***p* < 0.01, ****p* < 0.001.

Supplementary Figure 4 IL25 promoted stemness *via* GLI1 accumulation in CRC cancer. **(A)** Quantitative analysis of the western blotting of Hedgehog signaling genes in LoVo cells following IL25 silencing. **(B)** Quantitative analysis of western blotting in HT-29 and SW620 cells treated with SMO inhibitor Vismodegib and GLI1 inhibitor GANT-58 following IL25 treatment. **(C)** Quantitative analysis of the expression levels of GLI1, p-AMPK, and AMPK were examined in HT-29 and SW620 cells. **(D)** Quantitative analysis of western blotting of GLI1, p-AMPK, and AMPK in SW620 cells treated with AMPK activator Metformin following IL25 treatment. **(E)** Frequency (right) and representative day-7 images (left) of adenomatous organoids from mice treated with SMO inhibitor Vismodegib, GLI1 inhibitor GANT-58, and AMPK activator Metformin following IL25 treatment. Data present as mean ± *SEM*; **p* < 0.05, ***p* < 0.01, ****p* < 0.001.

Supplementary Figure 5 IL25 activated Hedgehog signaling pathway *via* SHH-N upregulation in CRC cancer. **(A)** The expression levels of SHH and PTCH1 in HT-29 and SW620 cells treated with recombinant IL25 in a time-dependent manner by Western blotting. **(B, C)** Cholesterol synthesis-related genes expression in HT-29 and SW620 cells treated with recombinant IL25 by RT-PCR. **(D)** Total cholesterol content in SW620 cells treated with IL25. **(E)** Quantitative analysis of western blotting of GLI1, p-AMPK, and AMPK in SW620 cells treated with AMPK activator A769662 following IL25 treatment. **(F)** Silencing effect of various siRNA of PTCH1 in SW620 cells. **(G)** Hedgehog signaling pathway genes expression was detected by Western blotting in SW620 following PTCH1 silencing. Data present as mean ± *SEM*; **p* < 0.05, ***p* < 0.01, ****p* < 0.001.
