# Supplementary figures and images for: IL25 Enhanced Colitis-Associated Tumorigenesis in Mice by Upregulating Transcription Factor GLI1
Source: Front Immunol. 2022 Mar 14;13:837262. doi: 10.3389/fimmu.2022.837262 (PMC8963976; doi:10.3389/fimmu.2022.837262)

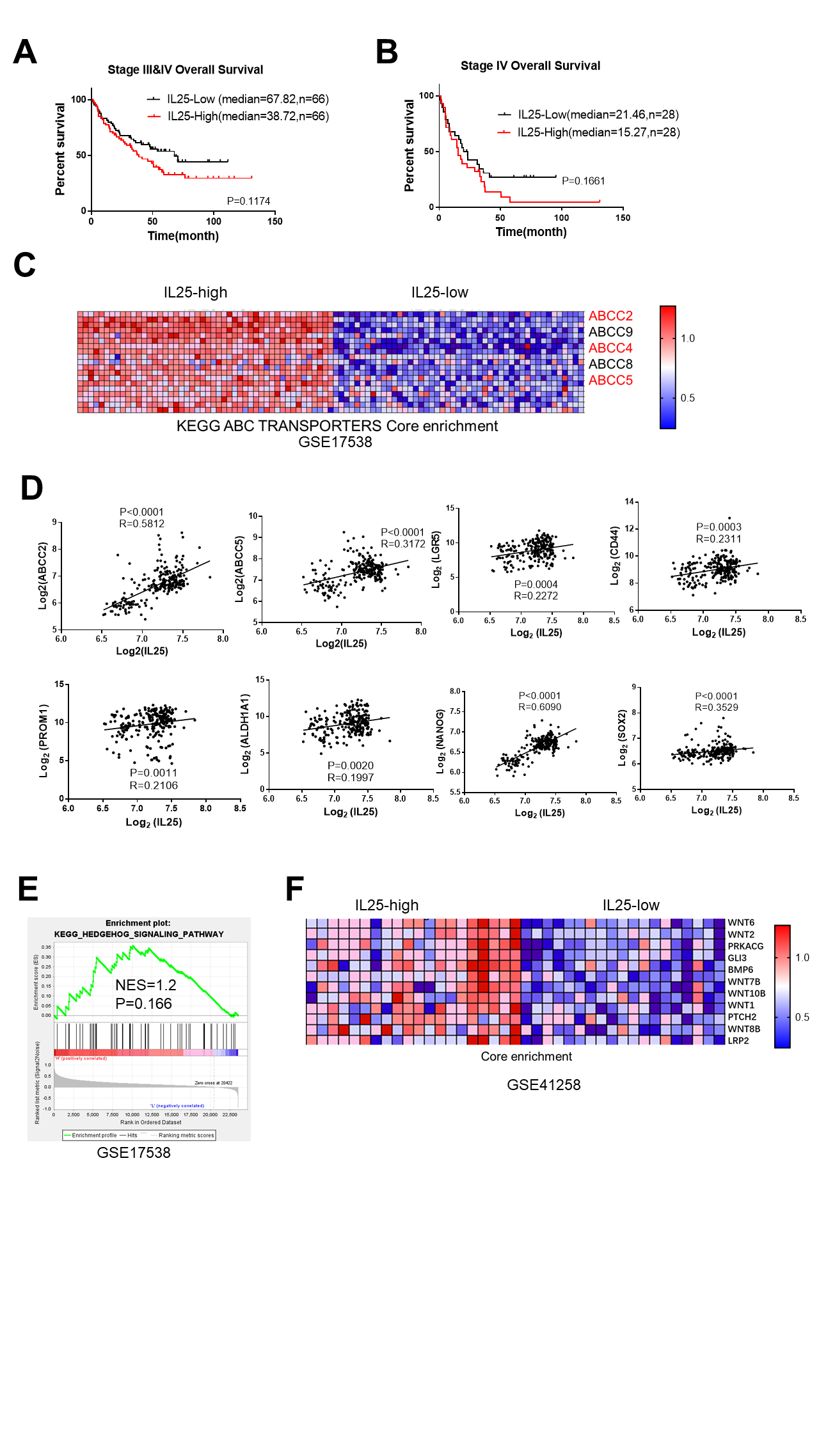

Supplement: Supplementary Figure 1 — GEO dataset analysis. (A, B) Overall survival analysis of CRC patients from GEO dataset GSE17538 in correlation with tumor IL25 expression. (C) Heat map showing core enrichment hallmark ABC transporters signaling genes in IL25 high expression colorectal cancer compared with IL25 low expression colorectal cancer from GSE17538. (D) Correlation analysis between IL25 and ABC transporters or stem cell markers from GSE17538. (E) Heat map showing core enrichment hallmark Hedgehog signaling genes in IL25 high expression colorectal cancer compared with IL25 low expression colorectal cancer from GSE41258. (F) GSEA for Hedgehog signaling pathway in IL25 high expression colorectal cancer compared with IL25 low expression colorectal cancer from GSE17538. [file Image_1.tif]

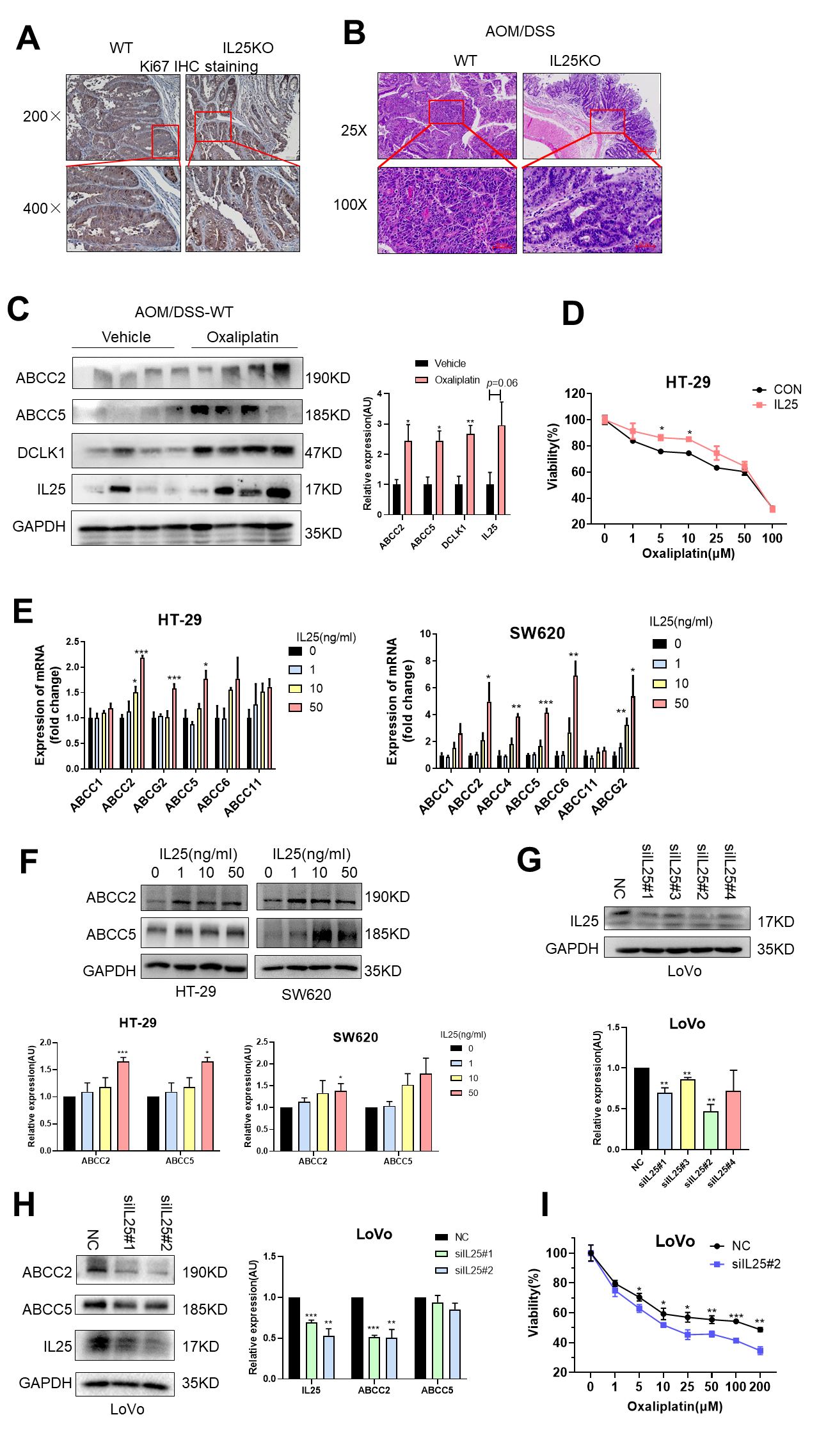

Supplement: Supplementary Figure 2 — IL25 expression was positively associated with chemotherapy drug transporters expression. (A) Representative images of Ki67 IHC staining. (B) Representative images of hematoxylin and eosin (H&E) from tumors in WT and IL25KO mice. (C) The expression levels of ABCC2, ABCC5, and IL25 were examined in tumor tissues by Western blotting. (D) HT-29 cells cultured with or without IL25 (50 ng/mL) for 24 hours and were subsequently exposed to oxaliplatin for 48 hours in a concentration-dependent. The cell viability was determined by the CCK-8 assay. (E, F) The expression levels of ABC transporters in HT-29 and SW620 cells treated with recombinant IL25 in a concentration-dependent manner by RT-PCR and Western blotting. (G) Silencing effect of various siRNA of IL25 in LoVo cells. (H) Western blotting of ABCC2 and ABCC5 in LoVo cells following IL25 silencing. (I) LoVo cells treated with oxaliplatin for 48 hours in a concentration-dependent following IL25 silencing. Data present as mean ± SEM; *p < 0.05, **p < 0.01, ***p < 0.001. [file Image_2.tif]

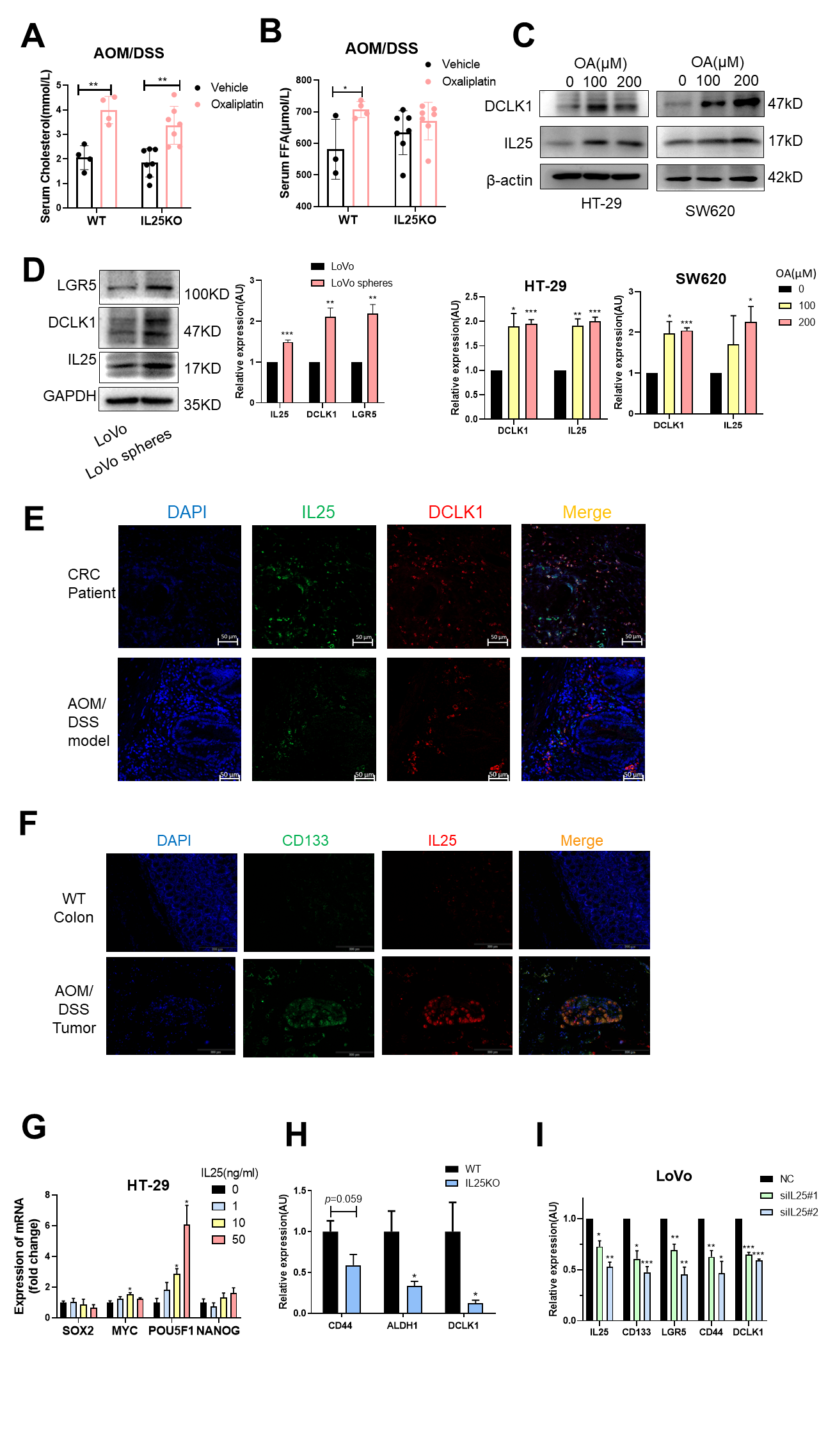

Supplement: Supplementary Figure 3 — IL25 was positively associated with stem cell genes expression. (A, B) Serum cholesterol and free fatty acid content in mice treated with the indicated treatment. (C) CRC cells were treated with 100 and 200μM oleic acid for 24h and cell lysates were analyzed by Western blotting with the indicated antibodies. (D) LoVo spheres were sorted from sphere formation assays by passaging spheres three times. LoVo cells and LoVo spheres lysates were analyzed by Western blotting. (E) IL25 and DCLK1 expression were detected by multiplexed fluorescence staining in the CRC patients and WT AOM/DSS-induced tumor tissues. The representative images show the expression of DCLK1 (red), DAPI (blue), IL25 (green). (F) IL25 and CD133 expression was detected by multiplexed fluorescence staining in the WT colon and WT AOM/DSS-induced tumor tissues. The representative images show the expression of IL25 (red), DAPI (blue), CD133 (green). (G) The expression levels of pluripotent stem cells markers, including NANOG, SOX2, MYC, POU5F1 were examined in HT-29 cells treated with recombinant IL25 in a concentration-dependent manner by RT-PCR. (H) Quantitative analysis of the western blotting of stem cells markers was examined in WT and IL25KO tumors. (I) Quantitative analysis of the western blotting of stem cells markers was examined in LoVo cells following IL25 silencing. Data present as mean ± SEM; *p < 0.05, **p < 0.01, ***p < 0.001. [file Image_3.tif]

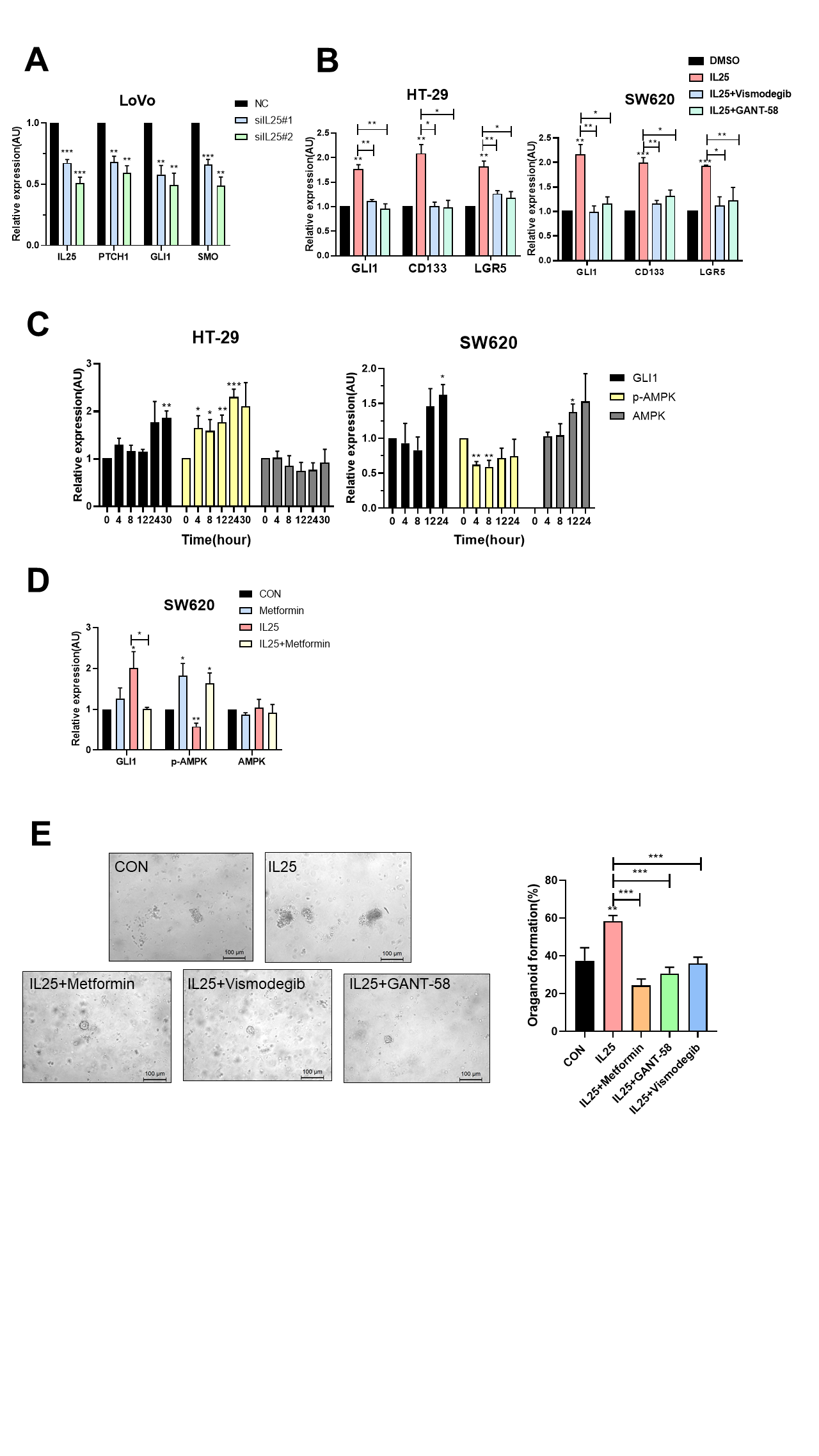

Supplement: Supplementary Figure 4 — IL25 promoted stemness via GLI1 accumulation in CRC cancer. (A) Quantitative analysis of the western blotting of Hedgehog signaling genes in LoVo cells following IL25 silencing. (B) Quantitative analysis of western blotting in HT-29 and SW620 cells treated with SMO inhibitor Vismodegib and GLI1 inhibitor GANT-58 following IL25 treatment. (C) Quantitative analysis of the expression levels of GLI1, p-AMPK, and AMPK were examined in HT-29 and SW620 cells. (D) Quantitative analysis of western blotting of GLI1, p-AMPK, and AMPK in SW620 cells treated with AMPK activator Metformin following IL25 treatment. (E) Frequency (right) and representative day-7 images (left) of adenomatous organoids from mice treated with SMO inhibitor Vismodegib, GLI1 inhibitor GANT-58, and AMPK activator Metformin following IL25 treatment. Data present as mean ± SEM; *p < 0.05, **p < 0.01, ***p < 0.001. [file Image_4.tif]

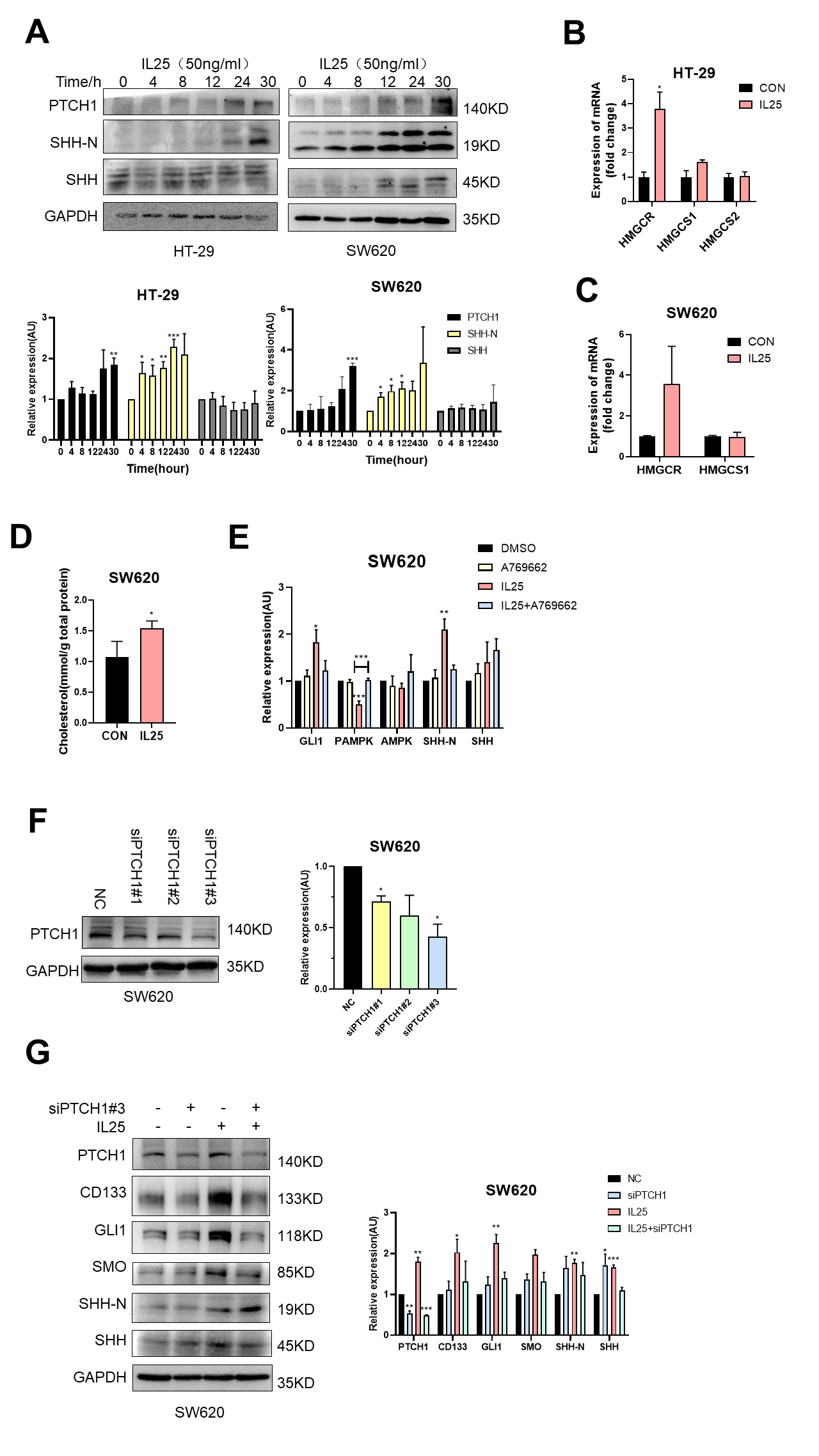

Supplement: Supplementary Figure 5 — IL25 activated Hedgehog signaling pathway via SHH-N upregulation in CRC cancer. (A) The expression levels of SHH and PTCH1 in HT-29 and SW620 cells treated with recombinant IL25 in a time-dependent manner by Western blotting. (B, C) Cholesterol synthesis-related genes expression in HT-29 and SW620 cells treated with recombinant IL25 by RT-PCR. (D) Total cholesterol content in SW620 cells treated with IL25. (E) Quantitative analysis of western blotting of GLI1, p-AMPK, and AMPK in SW620 cells treated with AMPK activator A769662 following IL25 treatment. (F) Silencing effect of various siRNA of PTCH1 in SW620 cells. (G) Hedgehog signaling pathway genes expression was detected by Western blotting in SW620 following PTCH1 silencing. Data present as mean ± SEM; *p < 0.05, **p < 0.01, ***p < 0.001. [file Image_5.tif]
