# Supplementary material for: IL25 Enhanced Colitis-Associated Tumorigenesis in Mice by Upregulating Transcription Factor GLI1
Source: Front Immunol. 2022 Mar 14;13:837262. doi: 10.3389/fimmu.2022.837262 (PMC8963976; doi:10.3389/fimmu.2022.837262)
Supplement: Supplementary file 6 [file DataSheet_1.docx]

Raw data:

https://www.jianguoyun.com/p/DU8z-j8Qov2TChiIwKQE
