# Supplementary material for: IL25 Enhanced Colitis-Associated Tumorigenesis in Mice by Upregulating Transcription Factor GLI1
Source: Front Immunol. 2022 Mar 14;13:837262. doi: 10.3389/fimmu.2022.837262 (PMC8963976; doi:10.3389/fimmu.2022.837262)
Supplement: Supplementary file 7 [file Table_1.docx]

**Supplementary** **Table.1 Correlation between expression of FUBP1 and clinicopathological features in 74 cases of CRC**

| Characteristics | No.of patients | Expression of IL-25 | | P-value |
| --- | --- | --- | --- | --- |
|  |  | Low | High |  |
| Patients |  |  |  |  |
| Maligant tumor | 74 | 54 | 20 |  |
| Adjacent tumor | 74 | 71 | 3 | <0.001 |
| Sex |  |  |  |  |
| Male | 44 | 32 | 12 |  |
| Female | 30 | 22 | 8 | 0.959 |
| Age |  |  |  |  |
| ＜60 | 28 | 22 | 6 |  |
| ≥60 | 44 | 31 | 13 | 0.446 |
| unknown | 2 | 1 | 1 |  |
| T classification |  |  |  |  |
| T1-T2 | 13 | 13 | 0 |  |
| T3-T4 | 57 | 37 | 20 | 0.012 |
| unknown | 4 | 4 | 0 |  |
| N classification |  |  |  |  |
| N0-N1 | 61 | 45 | 16 |  |
| N2-N3 | 13 | 10 | 3 | 0.813 |
| M classification |  |  |  |  |
| M0 | 65 | 47 | 18 |  |
| M1 | 9 | 7 | 2 | 0.729 |
| Differentiation |  |  |  |  |
| Well | 11 | 8 | 3 |  |
| Moderate/Poor | 63 | 46 | 17 | 0.984 |
| Differentiation |  |  |  |  |
| Well/Moderate | 58 | 43 | 15 |  |
| Poor | 16 | 11 | 5 | 0.667 |

**Supplementary** **Table.2 Primer sequence**

| Primer | Sequence（5’ to 3’） |
| --- | --- |
| HUMAN PTCH1 F | ACTTCAAGGGGTACGAGTATGT |
| HUMAN PTCH1 R | TGCGACACTCTGATGAACCAC |
| HUMAN HHIP F | TCTCAAAGCCTGTTCCACTCA |
| HUMAN HHIP R | GCCTCGGCAAGTGTAAAAGAA |
| HUMAN GLI3 F | GAAGTGCTCCACTCGAACAGA |
| HUMAN GLI3 R | GTGGCTGCATAGTGATTGCG |
| HUMAN GLI1 F | AGCGTGAGCCTGAATCTGTG |
| HUMAN GLI1 R | CAGCATGTACTGGGCTTTGAA |
| HUMAN GLI2 F | CATGGAGCACTACCTCCGTTC |
| HUMAN GLI2 R | CGAGGGTCATCTGGTGGTAAT |
| HUMAN SHH F | CCAAGGCACATATCCACTGCT |
| HUMAN SHH R | GTCTCGATCACGTAGAAGACCT |
| HUMAN IHH F | AGACCGCGACCGCAATAAG |
| HUMAN IHH R | GCCTTTGACTCGTAATACACCCA |
| HUMAN SMO F | GAAGTGCCCTTGGTTCGGA |
| HUMAN SMO R | GCAGGGTAGCGATTCGAGTT |
| HUMAN SUFU F | AGACCCCTTGGACTATGTTAGC |
| HUMAN SUFU R | CGAAGCTGATGTAGTGCCAGT |
| HUMAN ABCC5 F | AGTCCTGGGTATAGAAGTGTGAG |
| HUMAN ABCC5 R | ATTCCAACGGTCGAGTTCTCC |
| HUMAN ABCC6 F | AGATGGTGCTTGGATTCGCC |
| HUMAN ABCC6 R | GCCACACAGTAGGATGAATGAG |
| HUMAN ABCC1 F | CTCTATCTCTCCCGACATGACC |
| HUMAN ABCC1 R | AGCAGACGATCCACAGCAAAA |
| HUMAN ABCC11 F | CTTACGGAGTCGCTTAGATGAGA |
| HUMAN ABCC11 R | ATGCCCAGAAGTGCATCGAAA |
| HUMAN ABCC4 F | AGCTGAGAATGACGCACAGAA |
| HUMAN ABCC4 R | ATATGGGCTGGATTACTTTGGC |
| HUMAN WNT8A F | GAACTGCCCTGAAAATGCTCT |
| HUMAN WNT8A R | TCGAAGTCACCCATGCTACAG |
| HUMAN ABCC2 F | CCATAGCTTCATTCCTGAGTAGC |
| HUMAN ABCC2 R | TCAGAGGACGCTTGTAGCCTT |
| HUMAN ABCG2 F | CAGGTGGAGGCAAATCTTCGT |
| HUMAN ABCG2 R | ACCCTGTTAATCCGTTCGTTTT |
| HUMAN SOX2 F | TGGACAGTTACGCGCACAT |
| HUMAN SOX2 R | CGAGTAGGACATGCTGTAGGT |
| HUMAN LGR5 F | GAGTTACGTCTTGCGGGAAAC |
| HUMAN LGR5 R | TGGGTACGTGTCTTAGCTGATTA |
| HUMAN NANOG F | AAGGTCCCGGTCAAGAAACAG |
| HUMAN NANOG R | CTTCTGCGTCACACCATTGC |
| HUMAN PROM1 F | GGCCCAGTACAACACTACCAA |
| HUMAN PROM1 R | ATTCCGCCTCCTAGCACTGAA |
| HUMAN CD44 F | CTGCCGCTTTGCAGGTGTA |
| HUMAN CD44 R | CATTGTGGGCAAGGTGCTATT |
| HUMAN ALDH1A1 F | CTGCTGGCGACAATGGAGT |
| HUMAN ALDH1A1 R | CGCAATGTTTTGATGCAGCCT |
| HUMAN ACTB F | CATGTACGTTGCTATCCAGGC |
| HUMAN ACTB R | CTCCTTAATGTCACGCACGAT |
| HUMAN HMGCR F | TGATTGACCTTTCCAGAGCAAG |
| HUMAN HMGCR R | CTAAAATTGCCATTCCACGAGC |
| HUMAN HMGCS1 F | GATGTGGGAATTGTTGCCCTT |
| HUMAN HMGCS1 R | ATTGTCTCTGTTCCAACTTCCAG |
| HUMAN HMGCS2 F | GACTCCAGTGAAGCGCATTCT |
| HUMAN HMGCS2 R | CTGGGAAGTAGACCTCCAGG |
| HUMAN POU5F1 F | CAAAGCAGAAACCCTCGTGC |
| HUMAN POU5F1 R | TCTCACTCGGTTCTCGATACTG |
| HUMAN MYC F | GGCTCCTGGCAAAAGGTCA |
| HUMAN MYC R | CTGCGTAGTTGTGCTGATGT |
| MOUSE GLI1 F | CTCGACCTGCAAACCGTAATC |
| MOUSE GLI1 R | TCCTAAAGAAGGGCTCATGGTA |
| MOUSE GLI2 F | ACCCCTGATCCAGCCTTCA |
| MOUSE GLI2 R | GTTGGCATCATTTAGACAGTTGC |
| MOUSE DHH F | CTTGGCACTCTTGGCACTATC |
| MOUSE DHH R | CAGAGGCACAAGTTGCTTGC |
| MOUSE IHH F | CTCTTGCCTACAAGCAGTTCA |
| MOUSE IHH R | CCGTGTTCTCCTCGTCCTT |
| MOUSE GLI3 F | TGAGGGCCGTTACCATTATGA |
| MOUSE GLI3 R | GTCGGGCTACTAGATAAGGCA |
| MOUSE SHH F | AAAGCTGACCCCTTTAGCCTA |
| MOUSE SHH R | TTCGGAGTTTCTTGTGATCTTCC |
| MOUSE HHIP F | GGCTTCTACCCACGAGTATCT |
| MOUSE HHIP R | TGGAAGTGCTAGGTCCCCATC |
| MOUSE PTCH1 F | GCCTTCGCTGTGGGATTAAAG |
| MOUSE PTCH1 R | CTTCTCCTATCTTCTGACGGGT |
| MOUSE SMO F | CAATCGCTACCCTGCGGTTAT |
| MOUSE SMO R | CTGCTCGGCAAACAATCTCTC |
| MOUSE WNT1 F | TTCGGCAAGATCGTCAACCG |
| MOUSE WNT1 R | GCCAAAGAGGCGACCAAAATC |
| MOUSE Cd44 F | TGCAGGTATGGGTTCATAGAAGG |
| MOUSE Cd44 R | GTGTTGGACGTGACGAGGA |
| MOUSE Prom1 F | TGGGACTGCTGTTCATTATCCT |
| MOUSE Prom1 R | CATCTCTCCGCCGCATTTGT |
| MOUSE Lgr5 F | ACCTGTGGCTAGATGACAATGC |
| MOUSE Lgr5 R | TCCAAAGGCGTAGTCTGCTAT |
| MOUSE Nanog F | AGGACAGGTTTCAGAAGCAGA |
| MOUSE Nanog R | CCATTGCTAGTCTTCAACCACTG |
| MOUSE Pou5f1 F | CGGAAGAGAAAGCGAACTAGC |
| MOUSE Pou5f1 R | ATTGGCGATGTGAGTGATCTG |
| MOUSE Sox2 F | CGGCACAGATGCAACCGAT |
| MOUSE Sox2 R | CCGTTCATGTAGGTCTGCG |
| MOUSE Dclk1 F | CTGGGTTAATGATGATGGTCTCC |
| MOUSE Dclk1 R | TCCTGGTTGTTGGTAGTAGTCC |
| MOUSE Myc F | ATGCCCCTCAACGTGAACTTC |
| MOUSE Myc R | GTCGCAGATGAAATAGGGCTG |
| MOUSE ACTB F | GTGACGTTGACATCCGTAAAGA |
| MOUSE ACTB R | GCCGGACTCATCGTACTCC |

Supplementary Table.3 The siRNA sequences used for RNAi experiments are as follows:

| siRNA sequences | Sense(5'-3') | Antisense(5'-3') |
| --- | --- | --- |
| Homo- IL25#1 | CCAGGUGGUUGCAUUCUUGTT | CAAGAAUGCAACCACCUGGTT |
| Homo- IL25#2 | UCCUUAGCUUGUGUGUGUGTT | CACACACACAAGCUAAGGATT |
| Homo- PTCH1#1 | GCAGUGGAAAUUGGAACAUTT | AUGUUCCAAUUUCCACUGCTT |
| Homo- PTCH1#2 | GCAGACCAUGUUCCAGUUATT | UAACUGGAACAUGGUCUGCTT |
| Homo- PTCH1#3 | GGAGUUCACCGUUCACGUUTT | AACGUGAACGGUGAACUCCTT |
